# High-throughput RNAi screen for essential genes and drug synergistic combinations in colorectal cancer

**DOI:** 10.1038/sdata.2017.139

**Published:** 2017-10-03

**Authors:** Steven P. Williams, Andrew S. Barthorpe, Howard Lightfoot, Mathew J. Garnett, Ultan McDermott

**Affiliations:** 1 Wellcome Trust Sanger Institute, Hinxton CB10 1SA, UK

**Keywords:** Cell biology, Cancer

## Abstract

Metastatic colorectal cancer is a leading cause of cancer death. However, current therapy options are limited to chemotherapy, with the addition of anti-EGFR antibodies for patients with *RAS* wild-type tumours. Novel drug targets, or drug combinations that induce a synergistic response, would be of great benefit to patients. The identification of genes that are essential for cell survival can be undertaken using functional genomics screens. Furthermore, performing such screens in the presence of a targeted agent would allow the identification of combinations that result in a synthetic lethal interaction. Here, we present a dataset containing the results of a large scale RNAi screen (815 genes) to detect essential genes as well as synergistic combinations with targeted therapeutic agents using a panel of 27 colorectal cancer cell lines. These data identify genes that are essential for colorectal cancer cell survival as well as synthetic lethal treatment combinations using novel computational approaches. Moreover, this dataset could be utilised in combination with genomic profiling to identify predictive biomarkers of response.

## Background & Summary

Colorectal cancer is a leading cause of cancer mortality in the UK^1,2^, with >15,000 deaths each year. While early diagnosis leads to significantly higher survival, the prognosis for advanced stage disease remains poor. Therapeutic options for advanced colorectal cancer are currently limited to chemotherapy regimens (FOLFOX or FOLFIRI), and the anti-EGFR antibodies cetuximab or panitumumab^3,4^. While anti-EGFR therapy is beneficial in a subset of patients, tumours with activating *KRAS, NRAS* or *BRAF* mutations are intrinsically resistant, and exhibit constitutive activation of the MEK/ERK and/or PI3K/AKT signalling pathways^3,5,6^.

Despite the development of targeted agents over the last few decades, effective strategies to treat late-stage colorectal cancer have not emerged. While the concept of inhibiting a single signalling molecule remains attractive, in practice the inhibition of multiple targets often results in a more durable response. Furthermore, through the simultaneous inhibition of multiple signalling pathways, combination therapy may allow synthetic lethal interactions to be harnessed, and overcome intrinsic resistance to targeted agents^7–10^.

Several recent studies have successfully utilised high-throughput RNAi screening for profiling the essential gene landscape, and the identification of novel drug targets for cancer therapy^11–14^. Further, both chemical and genomic screens for synthetic lethal combinations have successfully identified targetable cancer-specific sensitivities^8,15–19^. However, it is important to note that in order to properly address the genomic heterogeneity of cancer, multiple cell lines are required. As is commonly seen, failure to do so results in a dataset that fails to capture the genetic diversity observed in the clinic, and identifies hits that are relevant only to the one cell line used.

**Figure 1:**
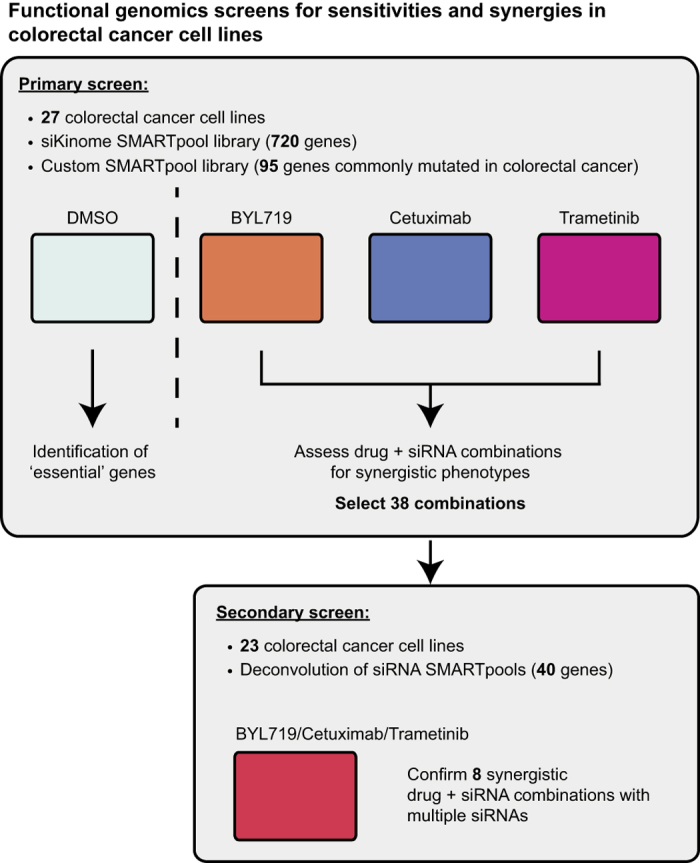
Overview of the successive functional genomics siRNA screens. The primary kinome siRNA screen was performed using a panel of 27 colorectal cancer cell lines. A DMSO-treated arm allowed identification of ‘essential’ genes, while the ‘plus-drug’ arms enabled selection of synergistic combinations for validation in the secondary screen.

In this study, high-throughput functional genomics screens were used to identify and validate synthetic lethal combinations in a panel of colorectal cancer cell lines. An overview of the experimental design is shown in Fig. 1. The screen contained four experimental arms: the DMSO anchor arm, to assess the activity of siRNA alone, and the three ‘plus-drug’ arms where cells were also treated with one of three anchor drugs targeting EGFR (cetuximab), MEK (trametinib) or PI3K (BYL719). Twenty-seven colorectal cancer cell lines were transfected with a customised siRNA library targeting the human kinome as well as 95 genes commonly mutated in colorectal cancer (Data records 1 and 3). We elected to target kinases in our screen as they are potentially more amenable to drug development in the event of specific vulnerabilities being identified. Additionally, for some kinases chemical probes or indeed drugs may already exist. Following 72 h incubation with the siRNA libraries, cells were assessed for decreased ATP levels (using the CellTiter Glo assay (Promega)), as an indicator of decreased viability.

The primary siRNA screen hits were binned according to the screen arm: DMSO arm hits were binned as ‘Essential’ using a z-score cut-off of <−3, while ‘plus-drug’ arm hits were ranked according to their synergy score, and the number of cell lines that showed a synergistic response (Data records 1 and 3). Essential genes identified in the primary screen were enriched for genes connected to the cell cycle, and contained many known pan-cancer single gene vulnerabilities^20–23^ (Data records 1 and 3). A total of 37 genes were identified that scored <−3 across three or more different cell lines.

The top 38 drug/gene synergistic combinations were selected for confirmation in a secondary deconvolution screen (4 siRNA sequences per gene) (Data records 2 and 3). siRNAs targeting the ‘Essential’ genes *PLK1* and *SF3B1* were also included. Deconvoluted siRNAs targeting 40 genes were rescreened as previously against the DMSO arm and three ‘plus-drug’ arms (Supplementary Fig. 1). This secondary screen led to the validation of 8 drug/siRNA combinations, which reproduced the original synergistic phenotype with 2 or more individual siRNA in greater than 75% of the cell lines that showed synergy in the original primary screen (Data records 2 and 3). These strict validation criteria mean that only hits that were confirmed in multiple cell line models with multiple siRNAs are considered ‘validated’ with high confidence and should be prioritised in future investigations. As a result the datasets presented here will be of benefit to the fields of cancer biology, therapeutics, and molecular signalling, and further provides the broader high-throughput screening community with a robust method and screening dataset.

## Methods

### Cell lines

A panel of commercially available colorectal cancer cell lines^24,25^ were grown in either RPMI or DMEM/F12 medium, supplemented with 10% fetal calf serum and 1% penicillin/streptavidin, and maintained at 37 °C in a humidified atmosphere at 5% CO_2_. Cell lines used in these screens, and the number of cells seeded per well, are listed in Table 1. The number of cells per well was established as part of the Genomics of Drug Sensitivity in Cancer (GDSC) screening project. The dynamic range within the assay was maximised by determining the number of cells required to achieve the greatest intensity value, while also ensuring the growth of untreated cells was unrestricted by factors such as space and nutrients. A minimum of 6 seeding densities were tested under screening conditions using a two-fold dilution series. All cell lines are routinely SNP profiled to detect cross-contamination and STR profiled to confirm their identity with the providing repository.

### Assay optimisation

Prior to screening we performed optimisation of assay conditions using a subset of 11 colorectal cancer cell lines. The type and concentration of transfection reagent was established by transfecting cells with Non-targeting siRNA pool #2 or si_PLK1 using six different transfection reagents (DharmaFECT 1, DharmaFECT 2, DharmaFECT 3, DharmaFECT 4 (all from Dharmacon), Lipofectamine RNAiMAX, and Lipofectamine 2,000 (both from Invitrogen)) at 4 different concentrations, with 4 different siRNA concentrations. The optimal transfection score was calculated for each cell line as follows:

$$OTS=Viability_{NT}\times \left(1–Intensity_{PLK}/Intensity_{NT}\right)$$

This score takes into account any negative effect on cell viability that the transfection reagent may have, as well as rewarding conditions where the positive control siRNA (si_PLK1) is best at reducing cell viability compared to the control. The conditions with the best average OTS across all 11 lines were selected for screening.

The concentration of anchor drugs was selected by performing dose response curves for each drug against 16 colorectal cancer cell lines. As the aim was to identify synergistic drug/siRNA combinations, it was important to select an anchor drug concentration that had a minimal effect on cell viability alone. A dose of the cetuximab and BYL719 compounds was therefore selected that inhibited cell viability by less than 20% in more than 75% of cell lines. Colorectal cancer is often driven by activation of the MEK/ERK pathway, and many of the lines on which trametinib was tested showed sensitivity to MEK inhibition. The dose of trametinib was therefore selected to 1) demonstrate effective inhibition of MEK phosphorylation and 2) inhibit cell viability by <30% in more than 50% of the cell lines.

### High throughput RNA interference screening

The protocol developed for this screen was adapted from previously published methods^26^, and is described below in detail. The primary siRNA screen was performed with biological replicates (see Supplementary Fig. 2 for number of replicates of each line), in 1,536-well plates. This enabled all 815 siRNA SMARTpools (4 siRNA pooled per gene) to be screened on one assay plate. A wide variety of positive and negative controls were located in specified wells across the plate, as depicted in Supplementary Fig. 1. The key positive controls for siRNA transfection were siRNA SMARTpools targeting *PLK1* (GE Dharmacon, #M-003290-01); known to be important for cell cycle, and a propriety cell death siRNA control (siTOX) (GE Dharmacon, #D-001500-01). The broad kinase inhibitor staurosporine (2 uM) was also added to select wells of each plate as a positive control for treatment by the anchor compounds. Non-targeting siRNA pool #2 was utilised as a negative control for siRNA transfection (GE Dharmacon, #D-001206-14). Mock transfected (lipid only) wells were also added as a reference. Other controls that were included, but not used were siRNA SMARTpool targeting KIF11 (GE Dharmacon, #M-003317-01), Non-targeting siRNA sequence #2 (GE Dharmacon, #D-001210-02) and siGENOME RISC-Free Control siRNA (GE Dharmacon, #D-001220-01).

The custom SMARTpool siRNA library was designed to target 95 genes commonly mutated in colorectal cancer (Data Record 3). Of these 95 genes, 15 were already present in the kinome library, resulting in 15 duplicates, and overall 794 unique siRNAs (the kinome library also has 6 duplicates). Note that duplicate wells are not able to be reported in Pubchem (Data Record 1), but are included in the raw data files (Data Record 3).

The siRNA transfer and addition of each anchor drug were performed using an Echo 555 acoustic dispenser (Labcyte Inc.). An XRD-384 (FluidX) automated reagent dispenser was used for all other liquid handling steps.

Specialised consumable reagents that were required to perform the siRNA screens:

- Primary screen: Dharmacon Human siGENOME SMARTpool siRNA Library—Protein Kinases (#G-003505-01, Lot 10,169), and a Dharmacon Human siGENOME Custom SMARTpool siRNA Library (see Data Record 3)
- Secondary screen: Dharmacon Human siGENOME Custom siRNA Library—Set of 4 siRNA duplexes per gene
- 1,536-well plates, polystyrene, tissue-culture treated, clear flat bottom wells, sterile, with lid, black (Corning Costar, Tewksbury, MA, #3893)
- Lipofectamine RNAiMAX Transfection Reagent (Invitrogen, #13778500)
- OPTI-MEM (Gibco, #31985062)
- CellTiter-Glo Luminescent Cell Viability Assay (Promega, #G7572)

### Day 1: siRNA reverse transfection

siRNA library and control plates were thawed at room temperature 1 h prior to use. Cells were incubated in TrypLE (Life Technologies) until detached, then washed in growth media, counted and diluted to the desired concentration in antibiotic-free DMEM/F12 or RPMI media (Table 1).

For the siRNA transfection, 70 nl of the siRNA library (2.5 μM stock) was dispensed into each well of 1,536-well plates (Corning, #3893) (using an Echo 555 acoustic dispenser). Lipofectamine RNAiMAX transfection reagent was diluted in OPTI-MEM media (1:50) and incubated for 5 min, before dispensing 1.5 μl into each well of each plate.

Plates were then incubated for 20 min at room temperature, before dispensing 6 μl cells to each well (FluidX XRD-384 multiwell dispenser) (total well volume 7.5 μl). The final siRNA concentration was 23.9 nM.

Column 1 of each plate received media only (no cells) for background luminescence readings. The plate layout was designed so that edge wells were not used, and control wells (positive and negative) were spread across the plate (Supplementary Fig. 1).

Plates were incubated at 37 °C in a humidified atmosphere at 5% CO_2_ in a Cytomat 24C rotating incubator (Thermo Fisher Scientific) to minimise temperature gradients.

### Day 2: Drug treatment

After 24 h incubation, 7.5 nl of anchor drug or vehicle (DMSO) alone was added to each well as appropriate. Cells were treated to achieve a final concentration of 5 μg ml^−1^ (32.9 μM) cetuximab (obtained from the Addenbrookes’ Hospital pharmacy), 10 nM trametinib (Selleckchem), or 1 μM BYL719 (Selleckchem). Vehicle treated wells received an equivalent volume of DMSO.

### Day 4: Viability assay

To measure cellular ATP levels, the CellTiterGlo assay (Promega) was used. 2.5 μl of CellTiterGlo reagent was added to each well and incubated for 10 min. Luminescence was then measured using a Molecular Devices Paradigm plate reader. Changes in ATP levels were used as an indicator of overall cell viability.

### Data analysis

The analysis of screen data entailed background correction, normalisation and scoring steps. Analysis was performed using custom R scripts, and is detailed below. Preliminary analysis of the raw data (Data Record 3) uncovered a relatively consistent diagonal viability gradient across each plate. The raw luminescence intensity readings for each plate of the primary screen were corrected for these position bias effects using a loess normalization approach^27,28^. This method was chosen as it performed better than the B-score method^29^, which we found to overfit the data and significantly increased the kurtosis of the dataset. Note that no correction method was required for the secondary screen data.

For each plate the luminescence intensity readings were background corrected by subtracting the mean value of blank wells. This removes any background noise that may result from the cell medium. Each well was then normalized to the mean of DMSO treated Non-targeting siRNA pool #2 negative control wells (24 wells) on that plate to obtain a relative viability score (Data records 1 and 3). Note that viability values were capped at a maximum of 1 in order to obtain meaningful Bliss additivity score values (below).

For quality control purposes, we calculated two Strictly Standardized Mean Difference (SSMD)^30–32^ values for each plate, using the two positive siRNA controls (siPLK1 and siTOX), and passed plates if either SSMD value was greater than 3^30^. Using plates that passed the SSMD threshold, the biological replicate plates for each cell line were then averaged.

In order to identify hits in the DMSO anchor arm (i.e., effect of siRNA alone) z-scores were calculated on a per-cell line basis^29^. Z-score normalisation is used to scale the results to a standard normal distribution, using the mean and standard deviation of the experimental wells. This approach ensured that variation in siRNA transfection efficiency across the panel did not affect our ability to select important viability genes in each cell line.

To identify synergistic drug/siRNA combination hits, we calculated the Bliss additivity score^33^ for each drug/siRNA combination across each cell line as following (where *V* is the observed relative viability):

$$Bliss\;additivity=1-\left(1-V_{siRNA}-1-V_{anchor}\right)+(\left(1-V_{siRNA})\times \left(1-V_{anchor}\right)\right)$$

We then calculated the synergy score for each combination, by computing the difference between the expected Bliss additivity and the observed viability of the combination as following:

$$Synergy\;score=Bliss_{combo}-V_{combo}$$

Each drug/siRNA combination was then ranked according to the number of cell lines where Synergy score > 0.15. We selected 38 top drug/siRNA combinations that showed strong synergy scores (Synergy score > 0.15), across three or more cell lines. We prioritised combinations that also resulted in lower overall viability. Many siRNAs that ranked highly with one anchor drug were also synergy hits with a second anchor, and so we designed the secondary screen so that all 38 siRNAs were rescreened against all three ‘plus-drug’ arms. The secondary screen utilised four siRNA sequences per gene, assayed separately (i.e., deconvoluted). The secondary screen data was then analysed using the same synergy score metric as previously, so that a synergy score was calculated for each individual siRNA duplex (Data records 2 and 3). For each siRNA duplex we determined whether the drug/siRNA combination reproduced a synergistic phenotype. The following threshold was used:

$$\begin{array}{l} If:Synergy_{primary}>0.15\;AND\;Synergy_{duplex}>\left(Synergy_{primary}-0.05\right),\\ \;then\;reproduced=TRUE. \end{array}$$

The number of siRNA duplex per cell line that scored as reproduced was then tallied for each combination.

## Data Records

### Data record 1

Primary siRNA screen data for all 27 cell lines are available at PubChem (Data Citation 1 to Data Citation 27). Assay ID accession numbers are provided in Table 2 (available online only). Screen-wide normalised data (negative control normalisation and z-score normalisation, where appropriate) are provided, as well as synergy scores for drug/siRNA combinations and the results of consequent binning strategies. The PubChem activity score indicates whether an siRNA was ‘active’ and binned as ‘Essential’ (designated 2, i.e., a screen hit in the DMSO arm) or ‘inactive’ (designated 1, i.e., not a screen hit). Samples are defined by siRNA catalogue number (Dharmacon) and Entrez Gene ID.

### Data record 2

Secondary deconvolution siRNA screen data for 23 cell lines are available at PubChem (Data Citation 28 to Data Citation 50). Assay ID accession numbers are provided in Table 2 (available online only). Screen-wide normalised data (negative control normalisation) are provided, as well as synergy scores for drug/siRNA combinations and the results of consequent binning strategies. The PubChem activity score indicates whether an siRNA was ‘active’ and binned as ‘Synergy (designated 2, i.e., a screen hit in any ‘plus-drug’ arm) or ‘inactive’ (designated 1, i.e., not a screen hit). Samples are defined by siRNA catalogue number (Dharmacon) and Entrez Gene ID.

### Data record 3

Raw data for both the Primary siRNA screen and the Secondary deconvolution screen are available at Figshare (Data Citation 51). Data for all assay plates are provided, including those with SSMD<3 that failed our QC threshold. Details of genes targeted by the custom siRNA library used in the Primary siRNA screen are also included, as are details of the siRNA/drug combinations selected for rescreening in the secondary deconvolution screen.

## Technical Validation

### Control performance and plate QC

**Figure 2:**
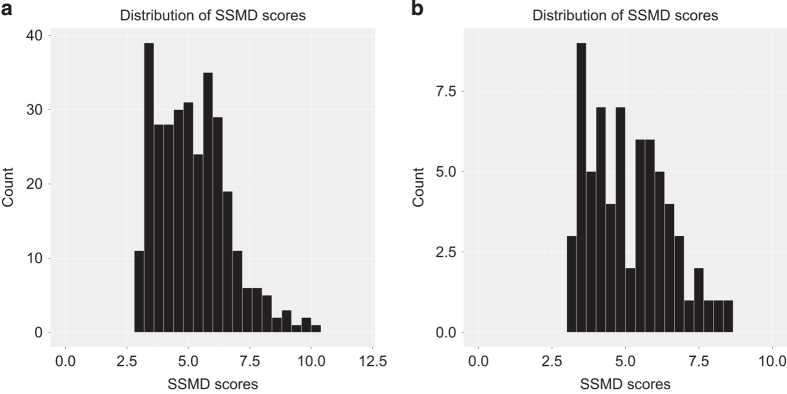
Distribution of plate SSMD scores. Histogram of SSMD scores that passed the quality control threshold SSMD >3 in the (**a**) primary screen, and (**b**) secondary screen.

The performance of positive and negative siRNA controls was quantified using the Strictly Standardised Mean Difference (SSMD)^30–32^ (Table 3); a statistical measure of the dynamic range between positive to negative controls, encompassing the mean and standard deviation of each control. Generally, a desirable SSMD for an RNAi screen is ≥3 in the context of a strong control^30–32^. We calculated two SSMD scores for each assay plate, using each of the positive siRNA controls (siTOX and siPLK1), compared with the negative control Non-targeting siRNA pool #2 (NTPool#2). While most cell lines responded equally well to both siTOX and siPLK1, there were some cell lines that did not respond to siTOX while siPLK1 had a large viability effect, and vice versa. Therefore, for each assay plate the higher of the two SSMD scores was used to pass or fail the plate. Plates that passed the quality control criteria had an average NTPool#2/siPLK1 SSMD of 4.23 and median of 4.09, and an average NTPool#2/siTOX SSMD of 4.12 and median of 4.16 (Table 3, Fig. 2) indicating very good data in the context of a strong positive control^27^.

Assay plates for four cell lines consistently failed (Supplementary Fig. 2), and could be attributed to very low siRNA transfection efficiency, or, in the case of MDST8, a significant unexplained viability decrease in the presence of the Non-targeting siRNA controls. These cell lines were therefore excluded from the primary screen. Of the 416 total plates set up for the primary screen, a total of 310 passed our QC threshold (74.5%). In the secondary deconvolution screen 83 plates were assayed, of which 71 passed the QC threshold (85.5%). Two cell lines (HCC-56 and SNU-407) could not be rescreened in the secondary screen due to technical reasons.

### Biological reproducibility across screening experiments

**Figure 3:**
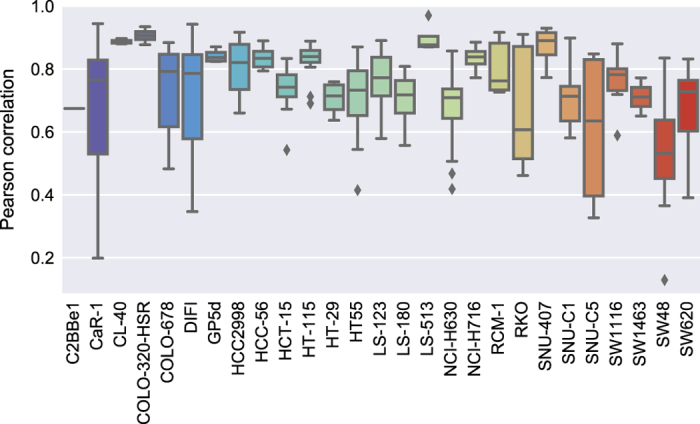
Distribution of Pearson correlation scores across biological replicate plates per cell line, from DMSO and synergy arms of primary screen.

To assess the biological replicate plate reproducibility, the Pearson correlation coefficient was calculated for each set of duplicate plates per cell line (using all plates from DMSO and synergy arms). All cell lines were highly reproducible with an overall median correlation between biological replicates of 0.77 (standard deviation: 0.13) (Fig. 3).

### Primary screen identified known candidates

**Figure 4:**
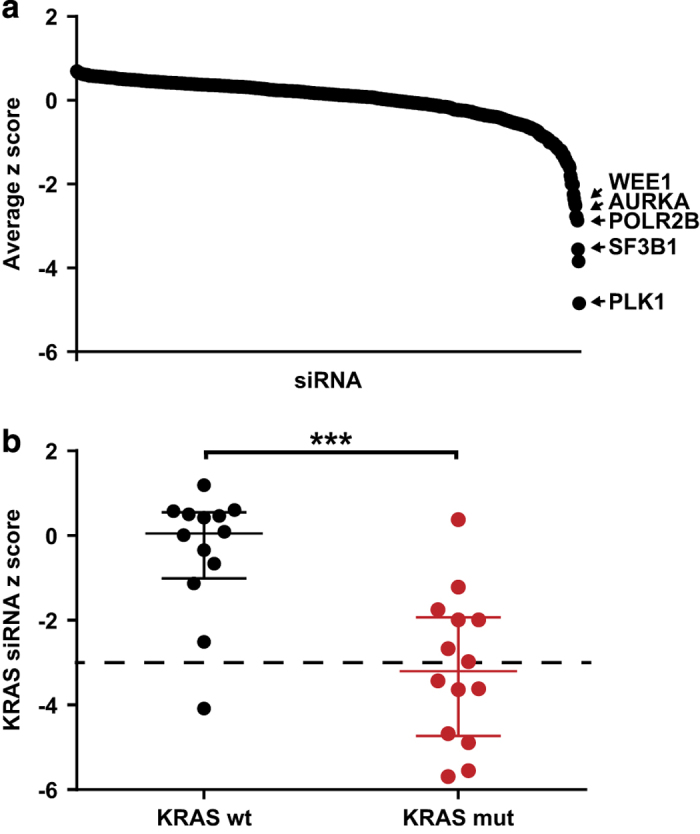
Essential colorectal cancer genes identified from the DMSO arm of primary screen. (**a**) The distribution of siRNAs according to the average z-score across the panel of cell lines. Hits with low scores correspond to known cell cycle and essential genes. (**b**) Plot showing the z-scores of *KRAS* siRNA in cell lines with *KRAS* wild-type status versus *KRAS* mutant status. ***P value<0.001.

The primary siRNA screen data (DMSO arm) returned a list of genes scored as essential (z-score<−3) for each cell line (Data record 1). Summarising this gene list showed that, as expected, *PLK1* (polo like kinase 1) was identified as essential (z-score<−3) in 24 of the 27 cell lines, with a further 2 lines scoring<−2.9. Other genes that were scored as essential in many of the cell lines include the cell cycle kinases *AURKA* (aurora kinase A) and *WEE1*, and *SF3B1* (mRNA splicing factor 3b subunit 1) (Fig. 4a).

Cancer cells that harbour mutations in the *KRAS* gene are expected to be highly dependent on KRAS expression compared to *KRAS* wild-type lines. siRNA-mediated knockdown of *KRAS* was found to be lethal (z-score<−3) for 7 of 14 *KRAS* mutant lines compared to 1 of 13 *KRAS* wild-type cell lines (mean z-score *KRAS* mutant=−3.12, mean *KRAS* wild-type=−0.37; *P*=0.0002) (Fig. 4b). While we focused on identifying essential gene phenotypes that were observed across multiple cell lines, our dataset also allows the identification of vulnerabilities that are restricted to a single cell line. One example is the sensitivity of NCI-H716 to siRNA knockdown of *FGFR2* (z-score=−4.31). Medico *et al.*^34^ recently reported that NCI-H716 has an amplification of *FGFR2* and is sensitive to an FGFR inhibitor as a result.

The analysis of the primary screen (synergy arms) synergy scores showed that the combination of siRNA targeting *PIK3CA* with the anchor drug trametinib was identified as having a synergistic phenotype (Synergy score>0.15) in 7 cell lines (Data record 1), in keeping with the role of PI3K signalling in resistance to MEK1/2 (*MAP2K1/2*) inhibition^35^. While the reverse combination (BYL719 anchor drug with siRNA targeting *MAP2K1*) was not identified as synergistic, this is likely due to redundancy between *MAP2K1* and *MAP2K2*. These results confirmed the ability of the screening methodology to identify known genes involved in colorectal cancer cell viability, and examples of synergistic drug/siRNA phenotypes.

### Number of combinations validated in the deconvolution screen

The deconvolution screen was designed to primarily confirm the top synergistic combinations. We did however also deconvolute siRNAs targeting the top essential genes *PLK1* and *SF3B1*. The results showed that individual siRNAs often produced stronger viability phenotypes than that observed using siRNA SMARTpools in the primary screen (Data record 2). In the Primary screen the effective concentration of each siRNA sequence in a SMARTpool is ~6 nM. However in the Secondary screen each individual siRNA sequence was 23.9 nM (4× higher concentration). While this may contribute to stronger viability phenotypes in the Secondary screen, part of the rationale for using siRNA SMARTpools is that by combining different siRNA sequences together the effective concentration of each siRNA sequence can be lower as the different siRNAs can act in concert.

The results of deconvoluted siRNAs validated the role of PLK1 (22/23 cell lines, where 2–4 of 4 siRNAs caused relative viability <0.5) and *SF3B1* (16/23 cell lines, where 2–4 of 4 siRNAs caused relative viability <0.5) as essential genes in colorectal cancer. Of note is the fact that several genes (e.g., *GUCY2D*, *CAMK2N1*) that were ranked high in the primary screen (DMSO arm) list of essential genes were included in the secondary screen due to their role in synergistic combinations. Neither of these two genes were convincingly validated as single gene vulnerabilities (*GUCY2D*: 1/23 cell lines, and *CAMK2N1*: 1/23 cell lines) (Data record 2), despite a recent report which also identified these genes as essential genes in a pan-cancer siRNA screen^11^. This highlights the importance of a secondary deconvolution stage in any siRNA screening campaign.

**Figure 5:**
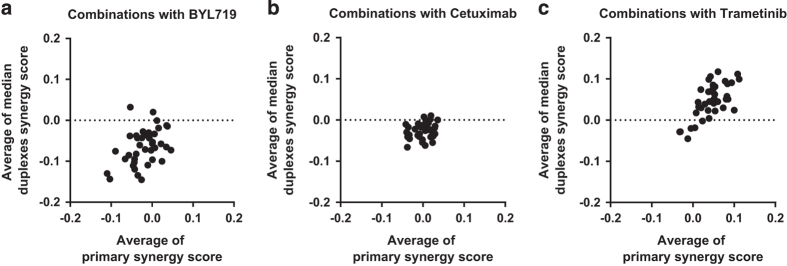
Reproducibility of synergy phenotypes between the primary and secondary screen. Plots of average synergy scores of siRNA combinations with (**a**) BYL719, (**b**) cetuximab and (**c**) trametinib anchor.

The secondary screen validated a large proportion of synergistic drug/siRNA combinations. Table 4 summarises the top drug/gene combinations validating in the deconvolution screen. Over 75% of synthetic lethal combinations tested reproduced the primary screen synergy effect with at least two siRNA duplexes in at least one cell line. Fourteen combinations were highly validated with at least 2 siRNA duplexes, in three or more cell lines. Eight combinations were validated with at least 2 siRNA duplexes, in >75% of lines that showed the phenotype in the primary screen. This included siRNA targeting *PIK3CA* in combination with the anchor drug trametinib. Overall, the average synergy score for each combination across all cell lines in the secondary screen was well correlated with the average synergy score for that combination in the primary screen (Fig. 5). Mechanistic characterisation of novel synergistic combinations will be the subject of further publications.

## Usage Notes

All siRNA screening data (Data records 1, 2 and 3) are provided so that users are able to investigate changes in viability and synergistic phenotypes by applying their own normalisation strategies and thresholds. This study focussed on identifying essential genes, across a heterogeneous panel of colorectal cancer cell lines. Genes that were scored as ‘Essential’ by siRNA knockdown alone (DMSO arm) can be investigated for their role in cell survival, with proof of principle being the lethality of *PLK1* knockdown. The dependence of *KRAS* mutant cell lines on expression of *KRAS*, further demonstrates that the dataset will reveal associations between gene essentiality and genomic biomarkers.

In addition, we investigated and validated a number of novel synergistic drug/gene combinations using multiple siRNAs and multiple colorectal cancer cell lines. Only a subset of candidate combinations from the primary screen (synergy arms) were rescreened, and as the dataset contains the overall viability and synergy scores for each siRNA pool, further instances of synthetic lethal combinations may be uncovered. While the aim of this study was to identify drug/siRNA combinations that showed a synergistic response, investigators may also want to analyse the overall cell viability measurements in order to identify effective combinations that yield high cell death. In the clinic these combinations could also have potential benefit for patients.

## Additional Information

**How to cite this article:** Williams, S. P. *et al.* High-throughput RNAi screen for essential genes and drug synergistic combinations in colorectal cancer. *Sci. Data* 4:170139 doi: 10.1038/sdata.2017.139 (2017).

**Publisher’s note:** Springer Nature remains neutral with regard to jurisdictional claims in published maps and institutional affiliations.

## Supplementary Material



Supplementary Information

## Figures and Tables

**Table 1:** Cell lines used in this study.

**Cell line**	**Cells/well (1,536-well plate)**	**Growth Media**
C2BBe1	400	D/F12
CaR-1	625	D/F12
CL-40	400	D/F12
COLO-320-HSR	625	RPMI
COLO-678	800	RPMI
DIFI	300	D/F12
GP5d	300	D/F12
HCC2998	400	RPMI
HCC-56	325	RPMI
HCT-15	150	RPMI
HT-115	400	D/F12
HT-29	200	RPMI
HT55	300	D/F12
HUTU-80*	400	D/F12
LS-123	250	D/F12
LS-180	150	D/F12
LS-513	300	RPMI
MDST8*	150	RPMI
NCI-H630	1,250	D/F12
NCI-H716	900	RPMI
RCM-1	400	RPMI
RKO	200	D/F12
SK-CO-1*	625	RPMI
SNU-407	400	RPMI
SNU-C1	625	RPMI
SNU-C2B*	500	RPMI
SNU-C5	325	RPMI
SW1116	625	D/F12
SW1463	400	D/F12
SW48	250	D/F12
SW620	350	D/F12

*Lines that failed QC in the primary screen. See Technical Validation, and Supplementary Fig. 2.

**Table 2:** Assay ID accession numbers.

**Cell line**	**Screen**	**AID**	**url**
C2BBE1	PRIMARY	1259276	https://pubchem.ncbi.nlm.nih.gov/bioassay/1259276
CAR1	PRIMARY	1259299	https://pubchem.ncbi.nlm.nih.gov/bioassay/1259299
CL40	PRIMARY	1259264	https://pubchem.ncbi.nlm.nih.gov/bioassay/1259264
COLO320HSR	PRIMARY	1259275	https://pubchem.ncbi.nlm.nih.gov/bioassay/1259275
COLO678	PRIMARY	1259281	https://pubchem.ncbi.nlm.nih.gov/bioassay/1259281
DIFI	PRIMARY	1259282	https://pubchem.ncbi.nlm.nih.gov/bioassay/1259282
GP5D	PRIMARY	1259267	https://pubchem.ncbi.nlm.nih.gov/bioassay/1259267
HCC2998	PRIMARY	1259261	https://pubchem.ncbi.nlm.nih.gov/bioassay/1259261
HCC56	PRIMARY	1259285	https://pubchem.ncbi.nlm.nih.gov/bioassay/1259285
HCT15	PRIMARY	1259300	https://pubchem.ncbi.nlm.nih.gov/bioassay/1259300
HT115	PRIMARY	1259283	https://pubchem.ncbi.nlm.nih.gov/bioassay/1259283
HT29	PRIMARY	1259291	https://pubchem.ncbi.nlm.nih.gov/bioassay/1259291
HT55	PRIMARY	1259298	https://pubchem.ncbi.nlm.nih.gov/bioassay/1259298
LS123	PRIMARY	1259266	https://pubchem.ncbi.nlm.nih.gov/bioassay/1259266
LS180	PRIMARY	1259279	https://pubchem.ncbi.nlm.nih.gov/bioassay/1259279
LS513	PRIMARY	1259289	https://pubchem.ncbi.nlm.nih.gov/bioassay/1259289
NCIH630	PRIMARY	1259286	https://pubchem.ncbi.nlm.nih.gov/bioassay/1259286
NCIH716	PRIMARY	1259260	https://pubchem.ncbi.nlm.nih.gov/bioassay/1259260
RCM1	PRIMARY	1259297	https://pubchem.ncbi.nlm.nih.gov/bioassay/1259297
RKO	PRIMARY	1259274	https://pubchem.ncbi.nlm.nih.gov/bioassay/1259274
SNU407	PRIMARY	1259280	https://pubchem.ncbi.nlm.nih.gov/bioassay/1259280
SNUC1	PRIMARY	1259290	https://pubchem.ncbi.nlm.nih.gov/bioassay/1259290
SNUC5	PRIMARY	1259306	https://pubchem.ncbi.nlm.nih.gov/bioassay/1259306
SW1116	PRIMARY	1259272	https://pubchem.ncbi.nlm.nih.gov/bioassay/1259272
SW1463	PRIMARY	1259259	https://pubchem.ncbi.nlm.nih.gov/bioassay/1259259
SW48	PRIMARY	1259284	https://pubchem.ncbi.nlm.nih.gov/bioassay/1259284
SW620	PRIMARY	1259307	https://pubchem.ncbi.nlm.nih.gov/bioassay/1259307
CAR1	SECONDARY	1259296	https://pubchem.ncbi.nlm.nih.gov/bioassay/1259296
CL40	SECONDARY	1259294	https://pubchem.ncbi.nlm.nih.gov/bioassay/1259294
COLO320HSR	SECONDARY	1259303	https://pubchem.ncbi.nlm.nih.gov/bioassay/1259303
COLO678	SECONDARY	1259295	https://pubchem.ncbi.nlm.nih.gov/bioassay/1259295
DIFI	SECONDARY	1259305	https://pubchem.ncbi.nlm.nih.gov/bioassay/1259305
HCC2998	SECONDARY	1259269	https://pubchem.ncbi.nlm.nih.gov/bioassay/1259269
HCT15	SECONDARY	1259287	https://pubchem.ncbi.nlm.nih.gov/bioassay/1259287
HT115	SECONDARY	1259304	https://pubchem.ncbi.nlm.nih.gov/bioassay/1259304
HT29	SECONDARY	1259288	https://pubchem.ncbi.nlm.nih.gov/bioassay/1259288
HT55	SECONDARY	1259278	https://pubchem.ncbi.nlm.nih.gov/bioassay/1259278
LS123	SECONDARY	1259277	https://pubchem.ncbi.nlm.nih.gov/bioassay/1259277
LS180	SECONDARY	1259301	https://pubchem.ncbi.nlm.nih.gov/bioassay/1259301
LS513	SECONDARY	1259262	https://pubchem.ncbi.nlm.nih.gov/bioassay/1259262
NCIH630	SECONDARY	1259273	https://pubchem.ncbi.nlm.nih.gov/bioassay/1259273
NCIH716	SECONDARY	1259293	https://pubchem.ncbi.nlm.nih.gov/bioassay/1259293
RCM1	SECONDARY	1259292	https://pubchem.ncbi.nlm.nih.gov/bioassay/1259292
RKO	SECONDARY	1259271	https://pubchem.ncbi.nlm.nih.gov/bioassay/1259271
SNUC1	SECONDARY	1259268	https://pubchem.ncbi.nlm.nih.gov/bioassay/1259268
SNUC5	SECONDARY	1259270	https://pubchem.ncbi.nlm.nih.gov/bioassay/1259270
SW1116	SECONDARY	1259302	https://pubchem.ncbi.nlm.nih.gov/bioassay/1259302
SW1463	SECONDARY	1259263	https://pubchem.ncbi.nlm.nih.gov/bioassay/1259263
SW48	SECONDARY	1259258	https://pubchem.ncbi.nlm.nih.gov/bioassay/1259258
SW620	SECONDARY	1259265	https://pubchem.ncbi.nlm.nih.gov/bioassay/1259265

**Table 3:** Summary of SSMD statistics.

	**NTpool#2/siTOX**	**NTpool#2/siPLK1**	**Best performing control**
	**SSMD**	**SSMD**	**SSMD**
siRNA primary screen			
Min	0.082621193	0.94572981	3.029108529
Max	10.04010781	8.970818571	10.04010781
Mean	4.121445128	4.237438466	5.156151715
Median	4.061560457	4.099758482	4.965521503
s.d.	2.01821525	1.618176452	1.45334267
siRNA secondary screen			
Min	0.768570773	1.013396597	3.145574494
Max	8.352036863	7.558236662	8.352036863
Mean	4.437169774	3.95085885	5.13568941
Median	4.540090885	3.830136971	4.962087199
s.d.	1.812114249	1.121988317	1.349978166

**Table 4:** Top validated synergistic drug/siRNA combinations.

**Combination (siRNA+anchor)**	¼*	**2/4**	**3/4**	**4/4**	**Total number of lines medium/ high validation**†	**Total number of lines high validation**	**Total lines with synergy in primary screen (rescreened in secondary)**	**% Validation**
PINK1 Trametinib	0	3	3	0	6	3	6	100
CRIM1 Trametinib	0	2	1	2	5	3	5	100
PIK3CA Trametinib	0	2	1	0	3	1	3	100
HUNK Trametinib	0	2	2	1	5	3	6	83.3
PIM1 Trametinib	0	3	2	0	5	2	6	83.3
CDKN2D Trametinib	0	1	0	3	4	3	5	80
BRAF Trametinib	0	1	2	0	3	2	4	75

*Number of cell lines where the synergistic phenotype was reproduced by 1 of 4 siRNA duplexes.

^†^medium=2/4, high=3/4 or 4/4.
