# Corrigendum: High-throughput RNAi screen for essential genes and drug synergistic combinations in colorectal cancer

**DOI:** 10.1038/sdata.2018.215

**Published:** 2018-10-09

**Authors:** Steven P. Williams, Andrew S. Barthorpe, Howard Lightfoot, Mathew J. Garnett, Ultan McDermott

Correction to: *Scientific Data* https://doi.org/10.1038/sdata.2017.139, published online 03 October 2017.

The Data Descriptor incorrectly states the formula used to calculate the Bliss additivity score in the Data analysis subsection of the Methods. The correct equation is listed below:

$$Bliss\;additivity=1–\left(1–V_{siRNA}\right)–\left(1–V_{anchor}\right)+\left(\left(1–V_{siRNA}\right)\times \left(1–V_{anchor}\right)\right)$$

