# Supplementary material for: High-throughput RNAi screen for essential genes and drug synergistic combinations in colorectal cancer
Source: Sci Data. 2017 Oct 3;4:170139. doi: 10.1038/sdata.2017.139 (PMC5625556; doi:10.1038/sdata.2017.139)
Supplement: Supplementary Information [file sdata2017139-s2.pdf]

Supplementary Figures

Figure S1. Plate maps for the siRNA library and controls in the (**a**) primary and (**b**) secondary screens.

Figure S2. Biological replicates per cell line. (**a**) Plot showing the number of primary screen biological replicate plates per cell line that were performed, and the number that passed quality control threshold SSMD > 3. Where the number of replicates attempted for a given cell line varied across the different experimental arms (e.g. for technical reasons), this is indicated by an asterisk and the number of replicates attempted for that arm given in brackets. (**b**) Plot showing the number of secondary screen biological replicate plates per cell line that were performed, and the number that passed quality control threshold SSMD > 3.

Figure S1

A

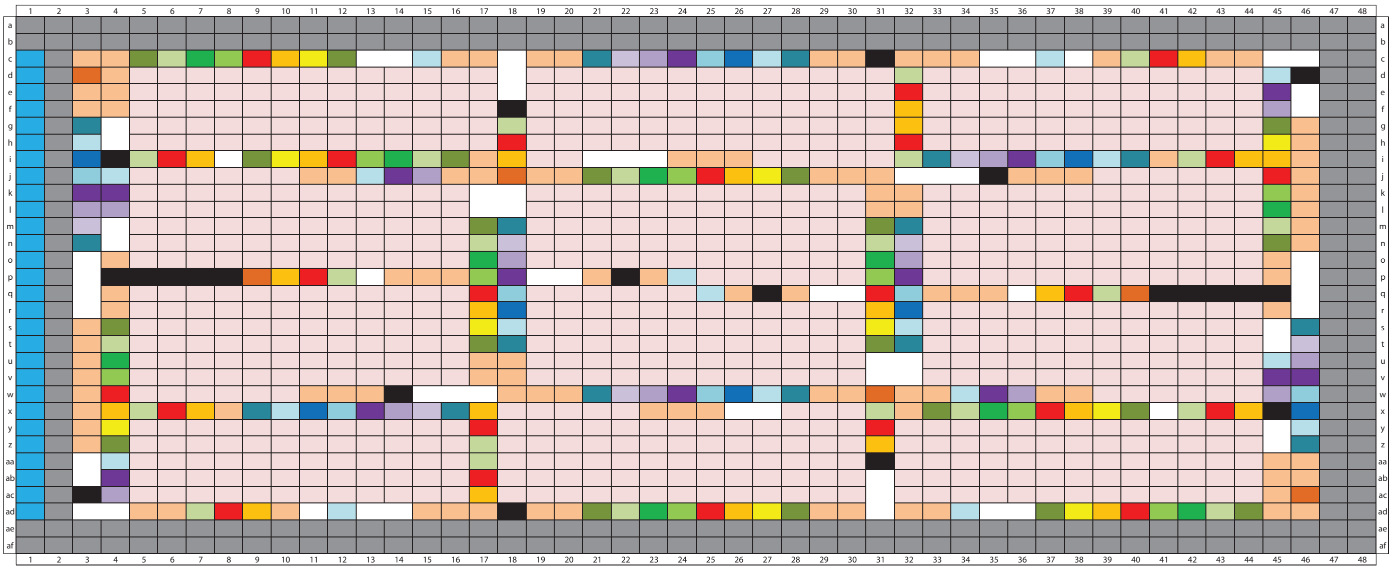

B	Blanks
UN-USED	Cells / Not used in analysis
	NC-0: Cells / No transfection reagent
	NC-1: Cells + transfection reagent only
	Staurosporine control
	siRNA library
	anchor drug only

Controls + DMSO	
C1	Mock
C2	NT SIRNA 2
C3	NT SIRNA POOL 2
C4	RISC FREE SIRNA
C5	siTOX
C6	siPLK1
C7	siKIF11
C8	Mock

Controls + anchor	
C1	Mock
C2	NT SIRNA 2
C3	NT SIRNA POOL 2
C4	RISC FREE SIRNA
C5	siTOX
C6	siPLK1
C7	siKIF11
C8	Mock

B

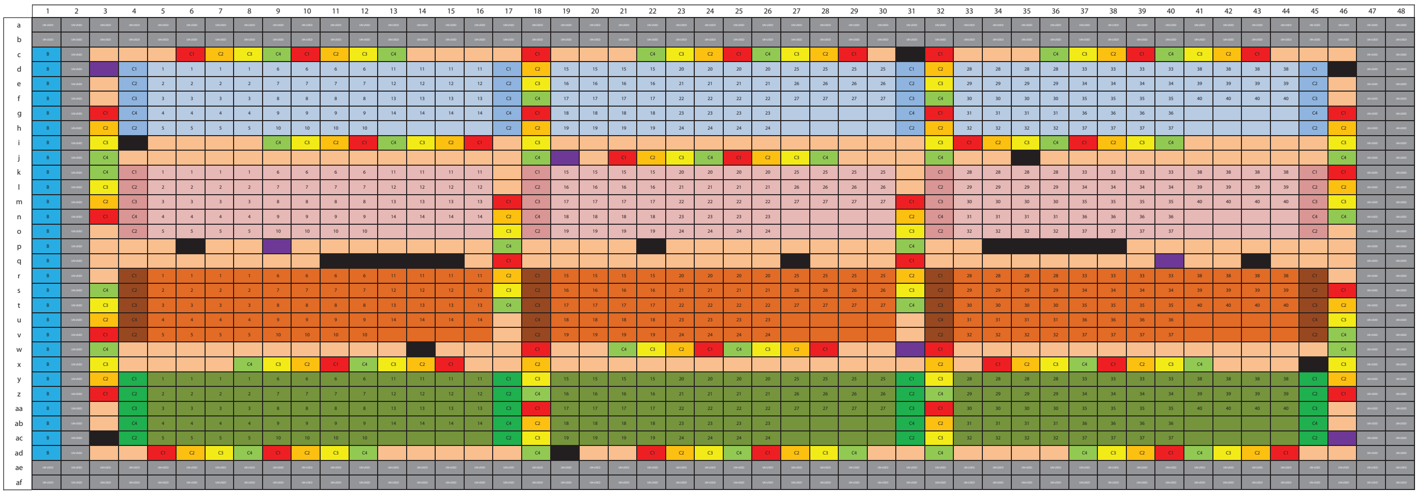

Controls	
C1	Mock
C2	NT#2
C3	PLK1
C4	siTOX

Anchors	
	DMSO
	BYL719
	Cetux
	Trametinib
	DMSO

B	Blanks
UN-USED	Cells / Not used in analysis
	NC-0: Cells / No transfection reagent
	NC-1: Cells + transfection reagent only
	Staurosporine control

Figure S2

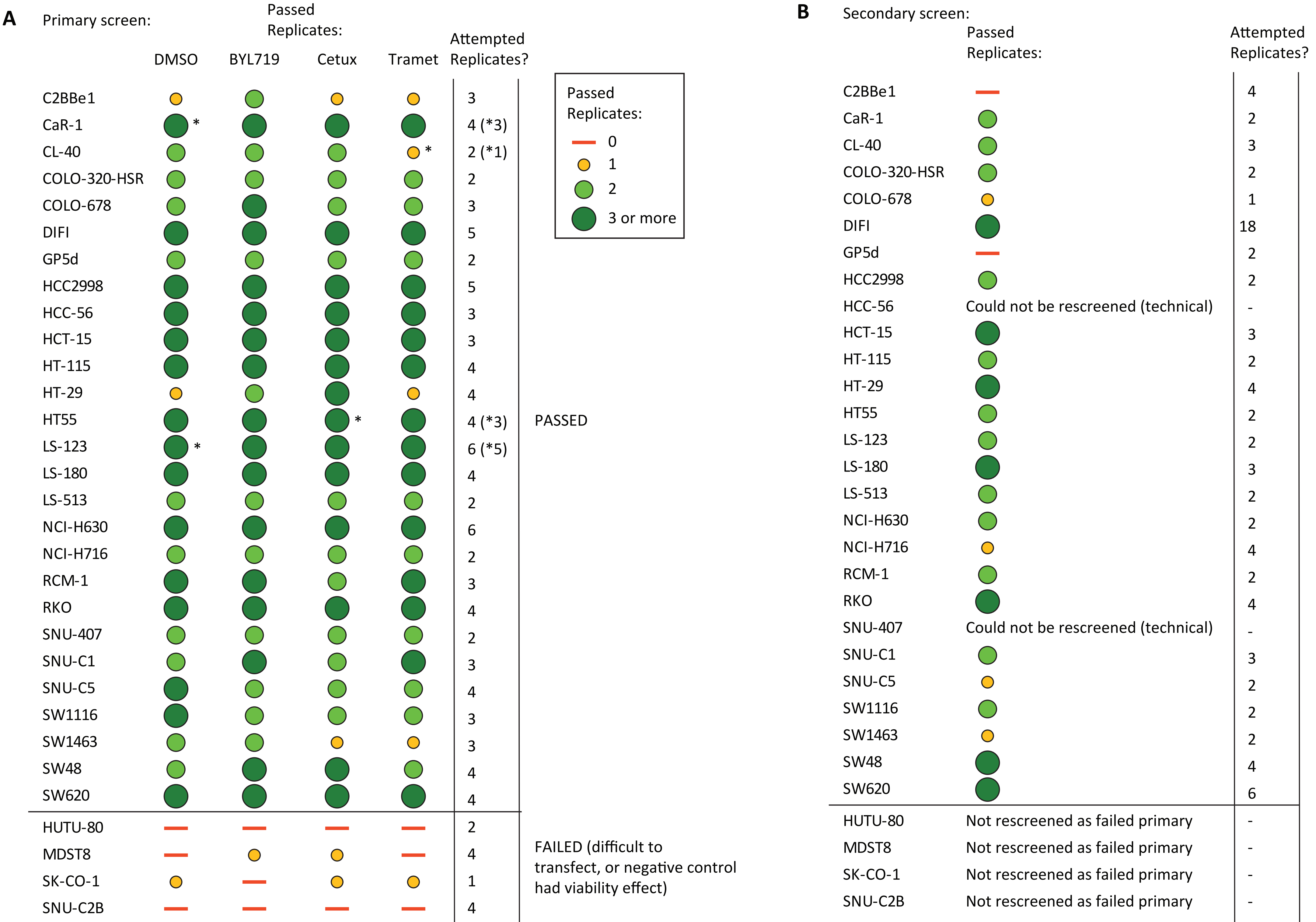
A
Primary screen:
Passed Replicates:
DMSO
BYL719
Cetux
Tramet
Attempted Replicates?
C2BBe1 3
CaR-1 * 4 (*3)
CL-40 * 2 (*1)
COLO-320-HSR 2
COLO-678 3
DIFI 5
GP5d 2
HCC2998 5
HCC-56 3
HCT-15 3
HT-115 4
HT-29 4
HT55 * 4 (*3)
LS-123 * 6 (*5)
LS-180 4
LS-513 2
NCI-H630 6
NCI-H716 2
RCM-1 3
RKO 4
SNU-407 2
SNU-C1 3
SNU-C5 4
SW1116 3
SW1463 3
SW48 4
SW620 4
PASSED
HUTU-80 2
MDST8 4
SK-CO-1 1
SNU-C2B 4
FAILED (difficult to transfect, or negative control had viability effect)
Passed Replicates:
0
1
2
3 or more
B
Secondary screen:
Passed Replicates:
Attempted Replicates?
C2BBe1 4
CaR-1 2
CL-40 3
COLO-320-HSR 2
COLO-678 1
DIFI 18
GP5d 2
HCC2998 2
HCC-56 Could not be rescreened (technical) -
HCT-15 3
HT-115 2
HT-29 4
HT55 2
LS-123 2
LS-180 3
LS-513 2
NCI-H630 2
NCI-H716 4
RCM-1 2
RKO 4
SNU-407 Could not be rescreened (technical) -
SNU-C1 3
SNU-C5 2
SW1116 2
SW1463 2
SW48 4
SW620 6
HUTU-80 Not rescreened as failed primary -
MDST8 Not rescreened as failed primary -
SK-CO-1 Not rescreened as failed primary -
SNU-C2B Not rescreened as failed primary -
